# Rare diseases: why is a rapid referral to an expert center so important?

**DOI:** 10.1186/s12913-023-09886-7

**Published:** 2023-08-23

**Authors:** Tina Willmen, Lukas Willmen, Anne Pankow, Simon Ronicke, Heinz Gabriel, Annette Doris Wagner

**Affiliations:** 1 Department of Nephrology, Hannover Medical School, Hanover, Germany; 2 Department of Prosthetic Dentistry and Biomedical Materials Research, Hannover Medical School, Hanover, Germany; 3 Department of Rheumatology and Clinical Immunology, Charité Berlin, Berlin, Germany; 4 Medical Clinic for Nephrology and Internal Intensive Care Medicine, Charité Berlin, Berlin, Germany; 5 Practice for Human Genetics Tübingen, Tübingen, Germany

**Keywords:** Rare diseases, Diagnostics, Prevention, Diagnostic odysseys, Target-oriented diagnostic procedures

## Abstract

**Background:**

Patients with rare diseases usually go through years of diagnostic odysseys. The large number of rare diseases and the associated lack of expertise pose a major challenge to physicians. There are few physicians dealing with patients with rare diseases and they usually work in a limited number of specialized centers. The aim of this study was to evaluate the diagnostic efficiency of an expert center.

**Methods:**

The diagnostic pathway of 78 patients of the outpatient clinic for rare inflammatory systemic diseases with renal involvement was analyzed retrospectively. For this purpose, each examination day was documented with the corresponding examinations performed from the onset of initial symptoms. Three time points were considered: The time when patients first visited a physician with symptoms, the time when patients consulted an expert, and the time when they received the correct diagnosis. In addition, it was documented whether the diagnosis could be made without the expert, or only with the help of the expert. The examinations that confirmed the diagnosis were also documented for each patient.

**Results:**

A correct diagnosis was made without the help of the expert in only 21% of cases. Each patient visited an average of 6 physicians before consulting the expert. Targeted diagnostics enabled the expert to make the correct diagnosis with an average of seven visits, or one inpatient stay. However, referral to the expert took an average of 4 years.

**Conclusion:**

The data show that rapid and targeted diagnostics were possible in the expert center due to the available expertise and the interdisciplinary exchange. Early diagnosis is of great importance for many patients, as an early and correct therapy can be decisive for the course of the disease.

**Supplementary Information:**

The online version contains supplementary material available at 10.1186/s12913-023-09886-7.

## Introduction

Patients with rare diseases go through a diagnostic odyssey of 4–5 years on average [1–3]. Considerably longer periods of 5–30 years are not uncommon [4].

Patients are often misdiagnosed during the course of their rare disease and, as a result, are incorrectly treated or, in some cases, undergo unnecessary surgery [5].

This is caused by many factors:

One main reason is the large number of about 8000 rare diseases [6, 7]. Due to their rarity, there are only a few experts who are familiar with these diseases.

Studies also show that the long road to correct diagnosis is an academic problem. Awareness of rare diseases is not sufficiently trained in medical education of physicians [8, 9].

However, this awareness is required to create the necessary starting point for a correct diagnosis. In cases of prolonged diagnostic uncertainty, lack of therapeutic success or atypical disease courses the initial suspicion of the presence of a rare disease is important to initiate a specific diagnosis and treatment of the patient [10].

Vandeborne et al. 2019 showed that general practitioners rated their knowledge of rare diseases as low [8]. Since they are the first point of contact for many patients, they are the starting point of the patient’s diagnostic wandering [11].

Patients’ frustration about the lack of successful treatment leads to frequent changes of physicians and further delays diagnosis, as the exchange of information among a large number of different physicians becomes increasingly difficult and diagnostic considerations always start from the beginning again [12–14].

Specialized centers for rare diseases have a high potential to shorten diagnostic odysseys or prevent them from arising in the first place. This can be attributed to the expertise and interdisciplinary exchange of many rare disease experts in the centers. Through their knowledge and awareness of rare diseases as well as access to innovative diagnostics [11], the experts can positively influence the diagnostic process.

The aim of this study was to demonstrate the benefits of rapid referral to expert centers using the Outpatient Clinic for Rare Inflammatory Systemic Diseases with Renal Involvement at the Hannover Medical School as an example. It was hypothesized that diagnosis by an expert with experience in rare diseases would be time-saving and effective.

## Methods

These data were collected as part of a retrospective study conducted at the Outpatient Clinic for Rare Inflammatory Systemic Diseases with Renal Involvement at Hannover Medical School [11, 15].

### The expert center

There are 37 clinics in whole Germany specialized in rare diseases - so-called A- centers. Hannover Medical School is one of them [16]. These A-centers represent a first point of contact for patients but also for treating physicians when a rare disease is suspected.

Patients with a suspected diagnosis are referred to specialized internal sub-centers, so-called B-centers, via the A-centers.

**Fig. 1:**
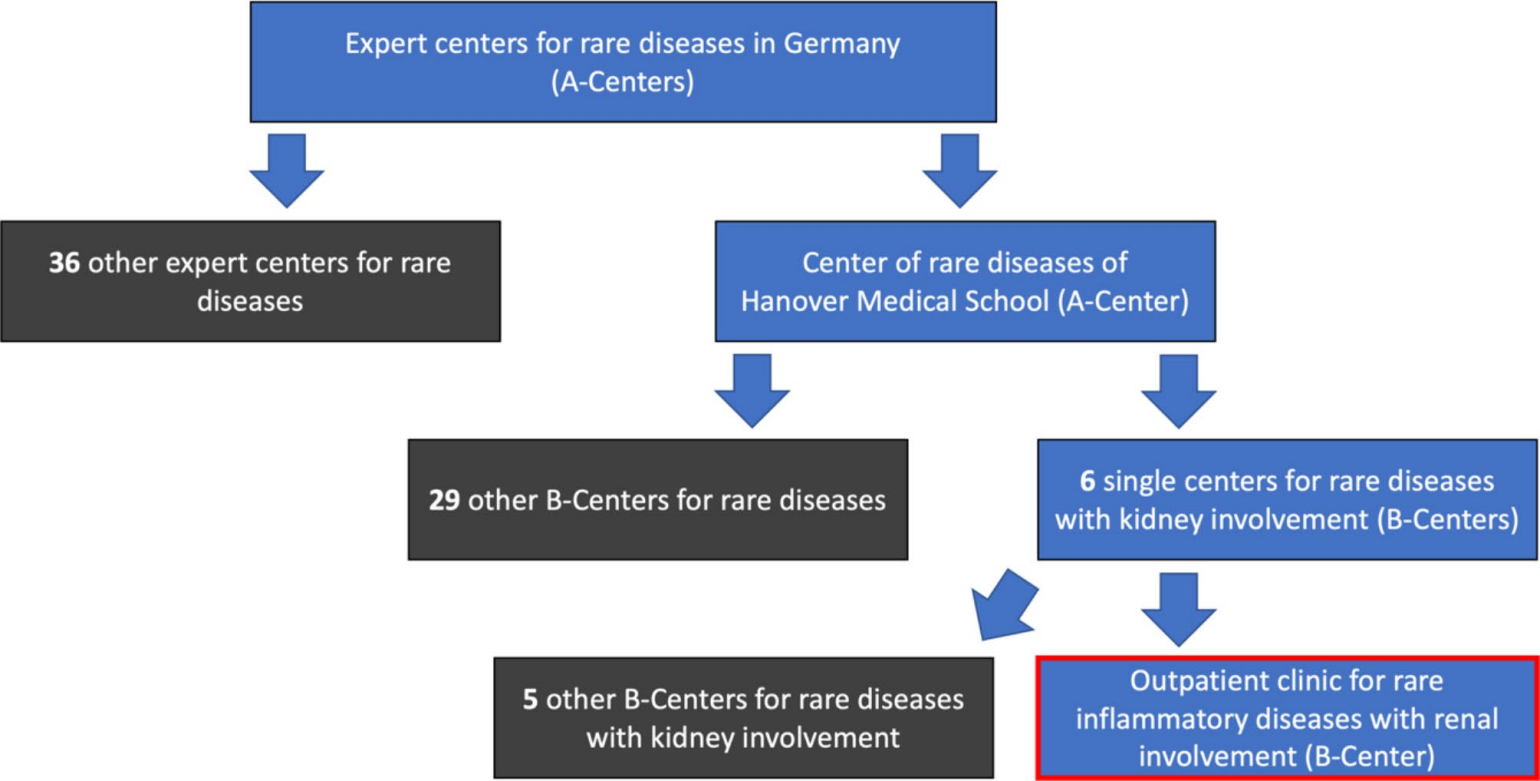
The figure shows the structure of centers for rare diseases in Germany [16, 17]. To illustrate the care function, we have marked the outpatient clinic for rare inflammatory systemic diseases with renal involvement in red

**Fig. 2:**
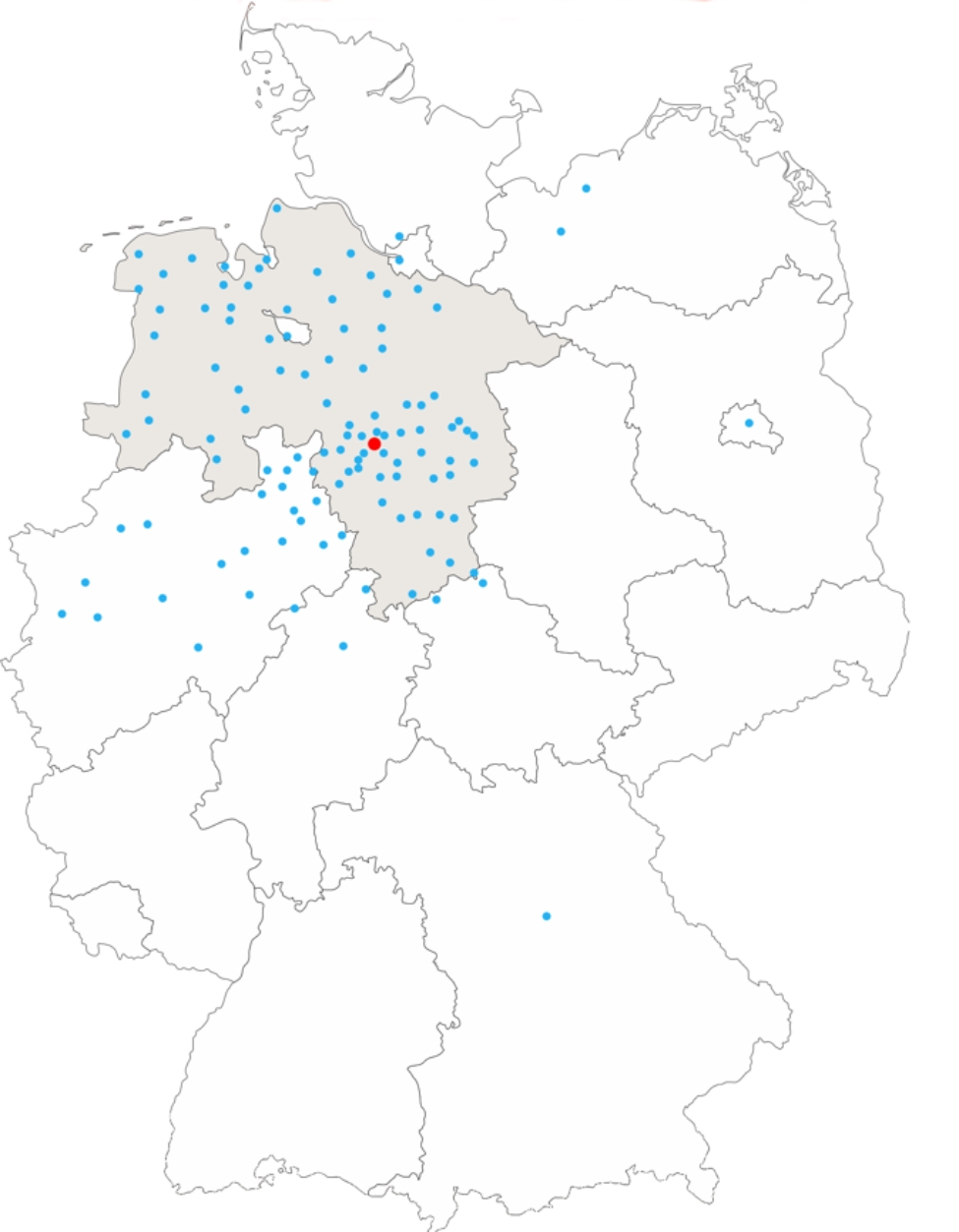
Overview of the catchment area of the outpatient clinic for rare inflammatory systemic diseases with renal involvement using the example of patient referrals in one year

At Hannover Medical School there are six specialized B-centers for nephrology alone [17]. One of these B-centers is the Outpatient Clinic for Rare Inflammatory Systemic Diseases with Renal Involvement (Fig. 1). The head of the outpatient clinic is a specialist in nephrology, rheumatology and laboratory medicine with many years of experience in rare diseases with renal involvement. Since there are only a few experts who are experienced in treating rare, inflammatory systemic diseases with kidney involvement, patients do not only come from the Hanover region, but from the northern part of Germany and even beyond (Fig. 2).

Currently, the outpatient clinic allocates between 1000 and 1100 appointments per year to patients. The number of new patients is 80 patients per year on average.

### Patient selection

The study includes 78 patients (Supplementary Table 1) with a clear definitive diagnosis by the year 2020. For therapy and monitoring, patients visit the outpatient clinic 2–4 times per year and there was no evidence of other suspected diagnoses since diagnosis.

The study participants represent a variety of rare inflammatory diseases with renal involvement.

The selection was made in part to cover the broad spectrum of different rare diseases diagnosed and treated at the outpatient clinic.

However, the most important point in the selection of the patient cases was a complete documentation of all physician visits during the course of the disease.

In the case of the patients examined, there was documentation of all visits to physicians with corresponding findings from physical and imaging diagnostics, but also from laboratory diagnostics.

### Procedure

For all patients, the onset of the first symptoms and the first visit to a physician were documented with the date.

From that point on, all data were noted if any form of diagnostic was performed. The exact form of imaging, laboratory, and physical diagnostics was documented. It was also noted whether the diagnosis was made by the expert or whether it had previously been made by outside physicians. In addition to the date of diagnosis the date of first presentation to the expert was recorded for patients whose disease could only be diagnosed by experts. Besides the number of days between the three points of time “first symptoms,“ “first presentation to the expert,“ and “final diagnosis,“ we also determined how many physician visits/hospitalizations occurred during the time leading up to diagnosis. Furthermore, it was determined for each patient how many different contact points with physicians and hospitals they had visited due to their illness. Descriptive statistics were used to examine the effect of referral to experts at the time of diagnosis.

In order to show the different approaches to diagnosis in an expert center and outside an expert center, the conducted examinations and investigations were considered separately. For each case, a list of investigations and examinations was made, which led to the confirmation of the final diagnosis. It was documented whether these examinations and investigations were performed outside the expert center, in the expert’s outpatient clinic, or upon the request of the expert. A comparative chart was prepared for the tests that most frequently led to confirmation of the diagnosis - organ biopsies, immunoserological markers, genetic testing and PET-CTs.

Excel was used for the calculation and Apple Numbers for the graphical presentation.

## Results

**Fig. 3:**
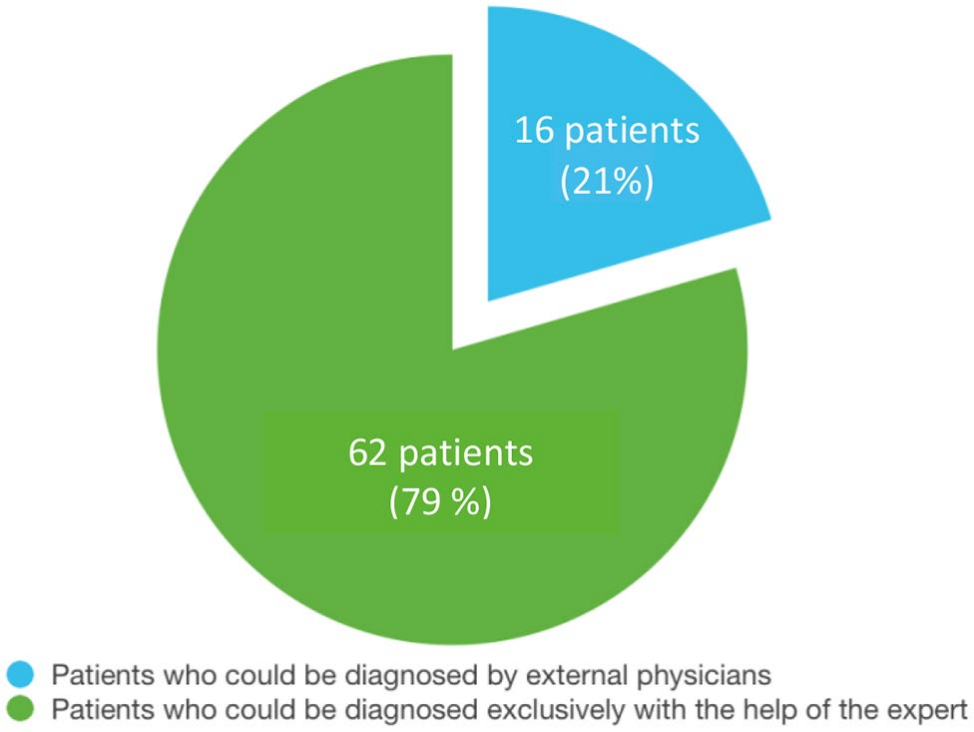
The diagram shows the distribution of patients (n: 78) who were diagnosed with the help of an expert and those who received a diagnosis from external physicians [15]

Out of 78 patients (Table 1) 16 patients could already be diagnosed before being referred to the expert (Fig. 3) [15]. This corresponds to 21% of all the patients. These patients were referred to the expert for further treatment.

**Table 1:** Characteristics of included cases [15]

	Female	Male	Total	
*Number of included cases*	48	30	78	
*Mean age at diagnosis*	43	46	44	
*Average number of different medical practices/hospitals visited*	7 (1 to 30)	6 (1 to 20)	6 (1 to 30)	
*Average number of years between the first visit to a physician and the diagnosis*	4 (0 to 28)	4 (0 to 29)	4 (0 to 29)	

On average, all study participants visited 6 physicians or hospitals before being referred to the expert [15].

**Fig. 4:**
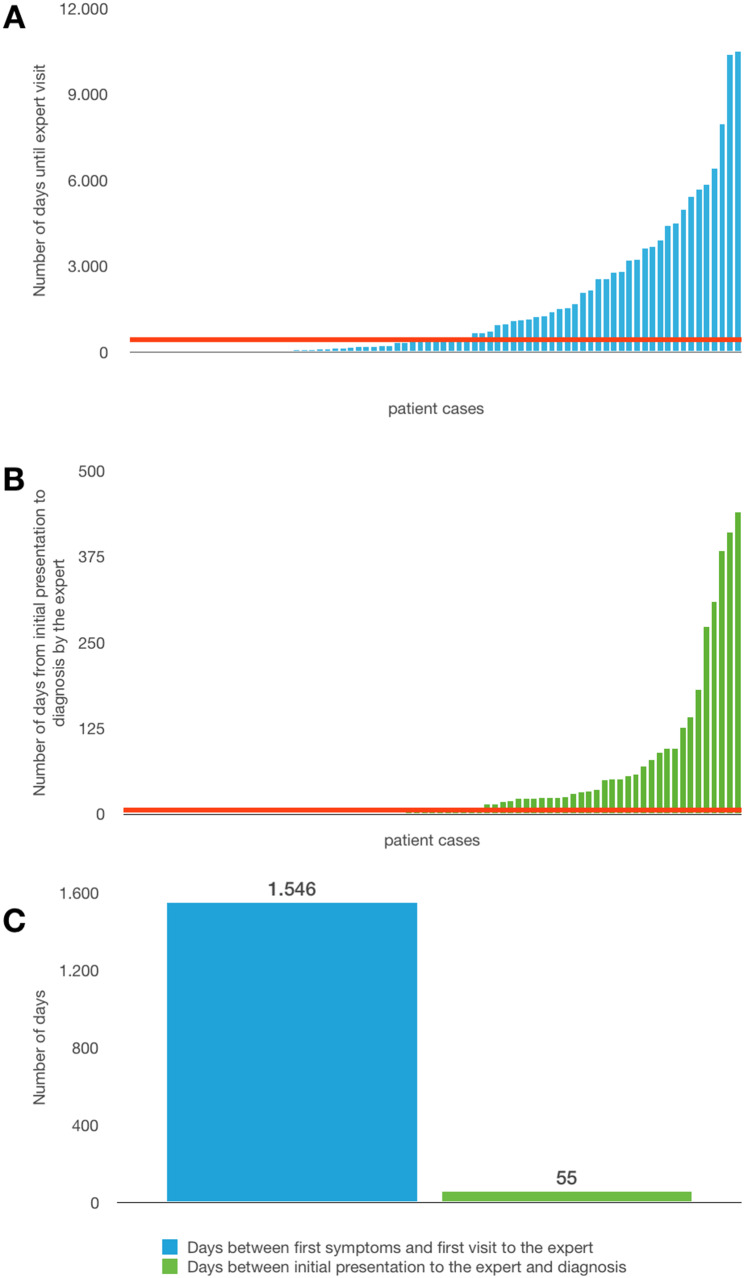
** A**: The graph shows for all cases (n:78) the number of days between first symptoms and presentation to the expert. The red line represents the median (421.5 days). The minimum was 0 days and the maximum was 10,496 days. **B**: The graph shows the number of days between the first visit to the expert and the diagnosis. The median here was 5.5 days (max: 439 days, min: 1 day). **C**: The graph shows a representation of the average values. On average, 1546 days passed before the expert visit and the expert needed an average of 55 days to make the correct diagnosis

An average of 4.4 years elapsed between the first visit to the physician and correct diagnosis. 1546 days passed approximately until a patient was referred to the expert. The average time from the first presentation to the expert to the day of confirmed diagnosis was 55 days (Fig. 4).

In the course of the individual diagnostic odysseys, an average of 30 physician appointments/hospitalizations for diagnostic tests took place.

Of these, an average of 7 examination days took place with the expert. Outside the expert center, an average of 25 examination appointments took place [15], but a significantly higher number of up to 109 examination appointments was also achieved in individual cases. Despite the significantly higher number of examination days, no diagnosis was made externally in 79% of the cases examined.

When analyzing the applied diagnostics that led to the confirmation of the diagnoses (Supplementary Table 2), four groups of the patient collective can be described:

1. Patients having already undergone the investigations essential for confirming the diagnosis, but where the correct interpretation of the results, due to lack of expertise, was only possible with the help of the expert (31 patients).
2. Patients diagnosed exclusively by the expert (29 patients).
3. Patients diagnosed outside the expert center- but initiated by the expert. Here cases were classified in which the expert recommended the targeted diagnostics as part of a medical consultation, or in which the expert followed up on previously performed physical examinations or investigations (2 patients).
4. Patients who had received both the target-oriented diagnostics and their correct interpretation outside the expert center (16 patients).

In the evaluated patient population, most diagnoses were clearly confirmed by immunoserological screenings, organ biopsies, genetic tests, and/or imaging - especially PET-CTs.

**Fig. 5:**
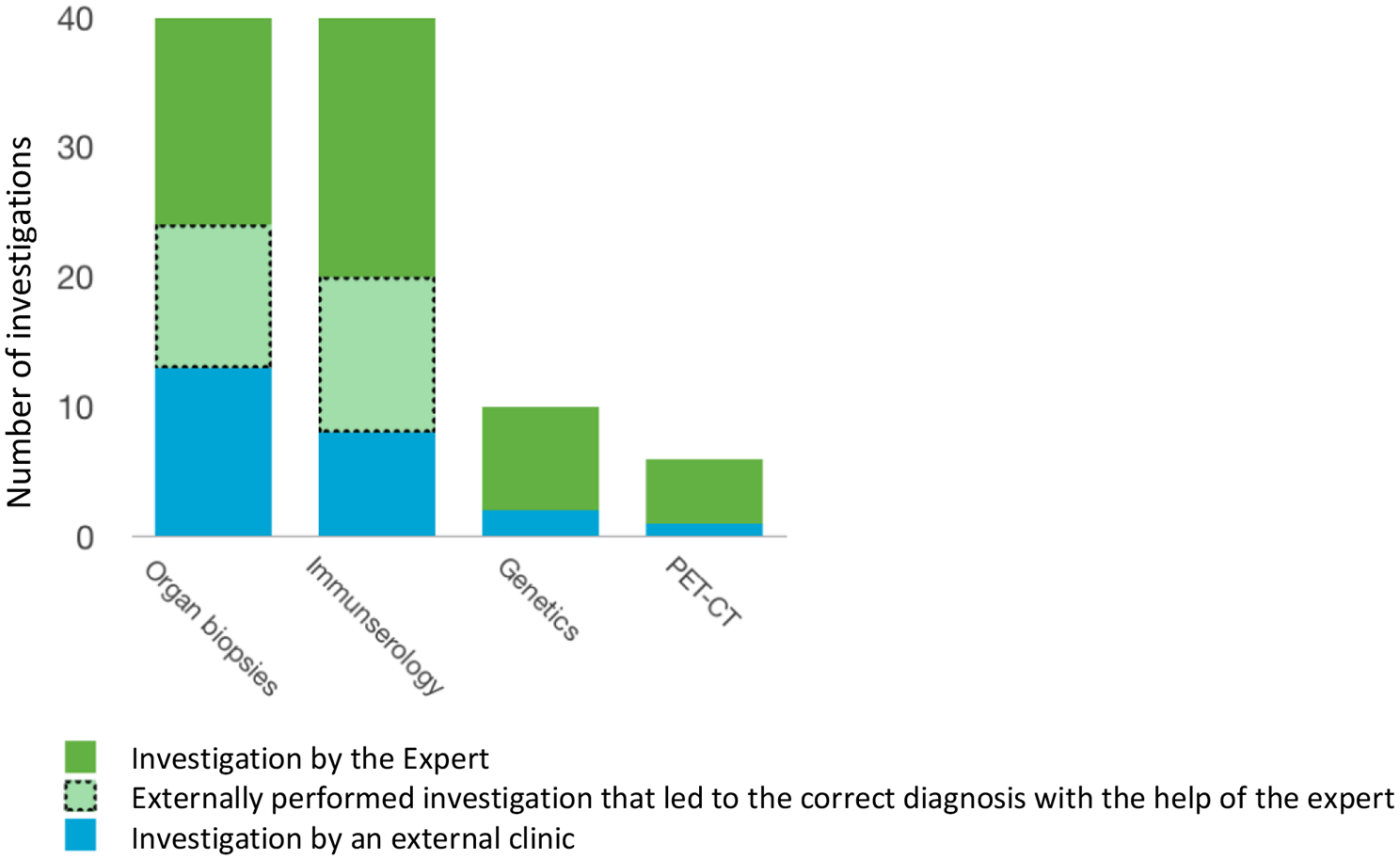
The graph shows the distribution of the investigations that are essential for the individual diagnoses. It shows who initiated the diagnostics that ultimately led to the correct diagnosis

The above-mentioned medical examinations and investigations were often applied or properly evaluated only by the expert (Fig. 5).

While the expert diagnosed a wide range of rare diseases efficiently, the diseases diagnosed outside the expert center tended to be life-threatening diseases. Granulomatosis with polyangiitis (GPA) is a typical example. In 7 of the total 16 externally diagnosed cases, GPA was diagnosed. The detection of anti-neutrophil cytoplasmic antibodies (ANCA) allowed the correct diagnosis to be made in the seven cases. But also, some genetic diseases, such as hereditary Mediterranean fever, or Fabry disease, could be diagnosed externally.

## Discussion

The results of our study show that the expertise and targeted approach of the expert lead to the correct diagnosis after a short time.

This suggests that rapid referral to an expert center can positively influence the diagnostic process. While the expert could make the correct diagnosis after an average of seven examination days, referral was often preceded by years of diagnostic uncertainty. In a study that included 375 patient interviews of vasculitis patients on diagnostic delays, Sreih et al., also concluded that rapid referral to experts can significantly shorten the diagnostic process [18]. The diagnostic benefit of referral to a specialized center is also demonstrated in a Dutch study by Mulders-Manders et al. [19]. The study showed that a diagnosis could be made in 110 of 193 referred patients with fever of unknown origin (FUO) by referral to an expert center.

The small number of examination days needed by the expert to make the correct diagnosis also confirms our hypothesis that the expert’s experience leads to a fast and efficient diagnosis. This positive effect on diagnostic yield is also mentioned in the Dutch study cited above. The authors write about a center that has had experience with FUO for 20 years and where only a small number of internists who frequently see patients with FUO treat them [19].

The analysis of the patient cases examined shows that many investigations, which were indispensable to ensure the correct diagnosis, were not properly evaluated or had not even been performed by the time of the presentation to the expert. These investigations mainly include organ biopsies, immune serological tests, PET-CTs and genetic testing.

Besides the expert know-how a good interdisciplinary exchange within the expert centers is also responsible for a good diagnostic efficiency. Experts from all disciplines work in the centers, and as rare diseases often affect multiple organ systems, it is important to assess them across disciplines.

Outside the expert center, patients lose a lot of time waiting for specialist appointments, taking up several weeks [20]. It is already known from other studies that ‘doctor hopping’, which is a consequence of the patients’ unsatisfactory health situation and the resulting loss of trust in the physicians treating them, also leads to delayed diagnosis [12, 13].

Examinations already conducted are often repeated when a physician is changed, and the diagnostic considerations of the previous physician get lost [12].

Often the number of different treating physicians and examinations already performed also complicate interdisciplinary work and thus the diagnostic process [13].

The patient population studied also reflects the following problem: the average patient saw six physicians before receiving the correct diagnosis, and the diagnostic process took an average of four years. Significantly longer diagnostic odysseys of up to 29 years were also recorded.

A major problem here is that late diagnoses of rare diseases often have irreversible consequences. Many rare diseases need to be diagnosed at an early stage to allow timely therapy without sequelae [21, 12]. This is also reflected in a patient example from the patient collective: a patient with IgG4-associated disease became blind in one eye due to late diagnosis. The eyesight of the other eye could be saved in time by the therapy of the expert.

In comparison to the cases where a late diagnosis results in a considerable damage to health, there are unfortunately also a number of rare diseases in which a late diagnosis leads to death [22].

The effects of a late diagnosis on the course of the disease are not the only consequences suffered by those affected.

Surveys also show the impact on the social and economic situation of patients with rare diseases:

For example, a recent study by Spencer-Tansley et al. in 2022 showed that 36% of the ill patients studied had suicidal thoughts [23]. Many suffer from anxiety, depression and/or stress due to their disease [23, 24]. 54% of patients surveyed in the USA also reported that their rare disease had led to isolation from friends [23]. Not only the patients themselves, but also relatives and caregivers are heavily burdened by the patients’ health situation [23, 25].

The economic impact of a rare disease and its delayed diagnosis should also not be forgotten. The patients often have limited or no ability to work, which can lead to a high financial burden. Often there are also health care costs that are not covered by the health insurance. In the USA, 37% of patients report having to borrow money from family or friends [24].

The health economic impact of early diagnosis should not be neglected either. An early and immediate diagnosis of a rare disease promises health economic savings [11].

### Limitations of the study

The study is limited by its retrospective design; therefore, the results are hypothetical and cannot be considered definitively proven.

To prove that a rapid referral to the expert center shortens the diagnostic process, a sufficiently large control group with externally made diagnoses would also have to be studied. A positive influence by the expert’s quick diagnosis can therefore be assumed, but not proven. It should also be taken into account that the expert only sees pre-selected cases due to her specialization and that this does not reflect the reality in medical practices. In addition, the expert has a better starting point due to the already existing study records.

As the study was based on collected patient records, there is some potential for error in the completeness of the records - particularly in the period before presentation to the expert, data may be missing despite careful checking for completeness.

Due to the broad spectrum of rare diseases studied, the results cannot be related to a single disease. The small number of patients suffering from a particular disease does not represent a sufficiently large reference value.

### Outlook

It would be of interest to investigate in prospective studies of rare diseases what factors might promote quick referral to expert centers:

The use of artificial intelligence seems promising. By using diagnostic decision support systems, physicians can optimize their diagnostics and become aware in time of the presence of rare diseases. Several authors have already been able to demonstrate the diagnostic benefit of such diagnostic decision support systems [26–28]. Especially for rare diseases, Ronicke et al. were able to prove a high potential of these systems [29]. Against this background a prospective study would be of interest that evaluates the impact on the diagnostic process of diagnostic decision support systems implemented in medical practices.

In an outlook on medicine in 2030, Hirsch et al. also report on the potential of symptom checker apps to alert patients to rare diseases [30].

In addition, it would be of interest to investigate the extent to which an increased education about rare diseases at medical and dental schools can raise awareness of rare diseases among physicians and dentists and thus have an impact on diagnostics.

## Conclusion

The study showed that after referral to an expert center, the correct diagnosis could be made in a comparatively short time of 55 days on average.

This suggests that rapid referral to an expert center can positively influence the diagnostic process.

The advantages of the expert center are the disease-specific experience of the experts, the good interdisciplinary exchange and the latest technical equipment in the expert centers, which allow the expert a different diagnostic procedure than in external practices.

### Electronic supplementary material

Below is the link to the electronic supplementary material.

Supplementary Material 1

Supplementary Material 2

## Data Availability

The data analyzed during this study are included in this published article and its supplemental information files. The data supporting the results of this study are available on request from the corresponding author ADW. The data are not publicly available because they contain information that could compromise study participant privacy and/or consent.

## Abbreviations

ANCA: Anti-neutrophil cytoplasmic antibodies
FUO: Fever with unknown origin
GPA: Granulomatosis with polyangiitis
PET-CT: Positron emission tomography–computed tomography
