# Supplementary material for: Rare diseases: why is a rapid referral to an expert center so important?
Source: BMC Health Serv Res. 2023 Aug 23;23:904. doi: 10.1186/s12913-023-09886-7 (PMC10463573; doi:10.1186/s12913-023-09886-7)
Supplement: Supplementary file 1 — Supplementary Material 1 [file 12913_2023_9886_MOESM1_ESM.docx]

**Supplementary Table 1: Case overview - diseases and the duration of the individual diagnostic odysseys**

| ***Case number*** | ***Diagnosis*** | ***Days from first symptoms to appointment with an expert*** | ***Days from initial presentation to diagnosis by the expert*** |
| --- | --- | --- | --- |
| **1** | IgG4- related disease | 4959 | 57 |
| **2** | Chronic sarcoidosis | 23 | 0 |
| **3** | Henoch Schonlein purpura (HSP) | 26 | 14 |
| **4** | Small fiber neuropathy | 2144 | 55 |
| **5** | Granulomatosis with polyangiitis (GPA) | 9 | 2 |
| **6** | Granulomatosis with polyangiitis (GPA) | 16 | 0 |
| **7** | Granulomatosis with polyangiitis (GPA) | 1116 | 0 |
| **8** | Takayasu’s arteritis | 70 | 1 |
| **9** | Giant cell arteritis | 26 | 8 |
| **10** | Granulomatosis with polyangiitis (GPA) | 123 | 0 |
| **11** | Granulomatosis with polyangiitis (GPA) | 72 | 0 |
| **12** | Granulomatosis with polyangiitis (GPA) | 10374 | 0 |
| **13** | Granulomatosis with polyangiitis (GPA) | 11 | 25 |
| **14** | Granulomatosis with polyangiitis (GPA) | 9 | 0 |
| **15** | Granulomatosis with polyangiitis (GPA) | 120 | 4 |
| **16** | Cryoglobulinemic glomerulonephritis in malignant disease | 11 | 22 |
| **17** | Cryoglobulinemia | 11 | 33 |
| **18** | Myositis associated lung disease with antisynthetase syndrome | 930 | 22 |
| **19** | Behcet’s disease | 2813 | 5 |
| **20** | Overlap Syndrom Primary biliary cholangitis and necrotizing myositis with cardiac involvement | 214 | 1 |
| **21** | Henoch-Schonlein purpura (HSP) | 1238 | 0 |
| **22** | Systemic lupus erythematosus (SLE) | 69 | 8 |
| **23** | Sjogren’s syndrome | 0 | 1 |
| **24** | Granulomatosis with polyangiitis (GPA) | 40 | 0 |
| **25** | Henoch-Schonlein purpura (HSP) | 45 | 0 |
| **26** | Sarcoidosis | 518 | 1 |
| **27** | Chronic polyarthritis and Familial Mediterranean fever (FMF) | 1219 | 14 |
| **28** | Whipple disease | 1531 | 8 |
| **29** | Takayasu’s arteritis | 83 | 1 |
| **30** | Polymyositis/scleroderma overlap | 2773 | 6 |
| **31** | Takayasu’s arteritis | 171 | 7 |
| **32** | Thromboangiitis obliterans, panarteritis nodosa | 12 | 79 |
| **33** | TNF receptor associated periodic syndrome (TRAPS) | 10496 | 24 |
| **34** | Familial Mediterranean fever (FMF) + Behcet’s disease | 2536 | 24 |
| **35** | Felty syndrome | 3904 | 273 |
| **36** | Spondyloarthritis | 1677 | 32 |
| **37** | Spondyloarthritis | 532 | 1 |
| **38** | Thrombotic thrombocytopenic purpura (TTP) | 1 | 5 |
| **39** | Cryoglobulinemia | 465 | 383 |
| **40** | IgG4-related disease | 4497 | 0 |
| **41** | IgG4-related disease | 164 | 19 |
| **42** | Familial Mediterranean fever (FMF) | 417 | 0 |
| **43** | systemic sclerosis, renal crisis | 35 | 0 |
| **44** | Antisynthetase syndrome | 4 | 18 |
| **45** | Antiphospholipid syndrome | 175 | 22 |
| **46** | Sarcoidosis | 7944 | 29 |
| **47** | IgG4-related disease | 380 | 1 |
| **48** | Hypophosphatasia | 3232 | 9 |
| **49** | Cryopyrin-associated periodic syndrome (CAPS) | 6412 | 181 |
| **50** | Gout in the spine and toe | 27 | 1 |
| **51** | Cryopyrin-associated periodic syndrome (CAPS) | 3684 | 1 |
| **52** | Panarteritis nodosa | 1378 | 1 |
| **53** | Spondyloarthritis | 670 | 3 |
| **54** | Fabry disease | 2063 | 0 |
| **55** | Fabry disease | 83 | 309 |
| **56** | Antisynthetase syndrome | 426 | 1 |
| **57** | CREST syndrome | 0 | 439 |
| **58** | Spondyloarthritis | 3181 | 49 |
| **59** | Relapsing Polychondritis | 0 | 89 |
| **60** | Behcet’s disease | 5663 | 126 |
| **61** | Chronic hepatitis C | 652 | 35 |
| **62** | Eosinophilic granulomatosis with polyangiitis (EGPA) | 5850 | 2 |
| **63** | Sjogren’s syndrome | 335 | 2 |
| **64** | Sjogren’s syndrome | 3626 | 141 |
| **65** | Stickler syndrome | 2554 | 410 |
| **66** | Spondyloarthritis | 345 | 1 |
| **67** | Focal segmental glomerulosclerosis (FSGS) | 321 | 51 |
| **68** | Tubulointerstitial nephritis and uveitis syndrome (TINU) | 1142 | 1 |
| **69** | Antisynthetase syndrome | 5433 | 51 |
| **70** | SAPHO syndrome | 409 | 0 |
| **71** | Polymyositiis | 14 | 1 |
| **72** | Chronic polyarthritis+ Sjogren’s syndrome | 1091 | 95 |
| **73** | Spondylarthritis | 203 | 95 |
| **74** | Cryoglobulinemia | 172 | 1 |
| **75** | Mixed amyloidosis | 974 | 0 |
| **76** | Renal cell carcinoma, Systemic lupus erythematosus (SLE), Antiphospholipid syndrome (APS) | 721 | 24 |
| **77** | SAPHO syndrome | 1510 | 69 |
| **78** | SAPHO syndrome | 4413 | 1 |
| Legend: |  |  |  |
|  |  |  |  |
|  | : Patients with external diagnosis | |  |
